# Fasentin diminishes endothelial cell proliferation, differentiation and invasion in a glucose metabolism-independent manner

**DOI:** 10.1038/s41598-020-63232-z

**Published:** 2020-04-09

**Authors:** Mª Carmen Ocaña, Beatriz Martínez-Poveda, Manuel Marí-Beffa, Ana R. Quesada, Miguel Ángel Medina

**Affiliations:** 1 Universidad de Málaga, Andalucía Tech, Departamento de Biología Molecular y Bioquímica, Facultad de Ciencias, E-29071 Málaga, Spain; 2 IBIMA (Biomedical Research Institute of Málaga), E-29071 Málaga, Spain; 3 Universidad de Málaga, Andalucía Tech, Departamento de Biología Celular, Genética y Fisiología, Facultad de Ciencias, E-29071 Málaga, Spain; 4 CIBER de Enfermedades Raras (CIBERER), E-29071 Málaga, Spain

**Keywords:** Tumour angiogenesis, Target identification

## Abstract

The synthetic compound fasentin has been described as a modulator of GLUT-1 and GLUT-4 transporters, thus inhibiting glucose uptake in some cancer cells. Endothelial glucose metabolism has been recently connected to angiogenesis and it is now an emerging topic in scientific research. Indeed, certain compounds with a known effect on glucose metabolism have also been shown to inhibit angiogenesis. In this work we tested the capability of fasentin to modulate angiogenesis *in vitro* and *in vivo*. We show that fasentin inhibited tube formation in endothelial cells by a mechanism that involves a negative effect on endothelial cell proliferation and invasion, without affecting other steps related to the angiogenic process. However, fasentin barely decreased glucose uptake in human dermal microvascular endothelial cells and the GLUT-1 inhibitor STF-31 failed to inhibit tube formation in these cells. Therefore, this modulatory capacity on endothelial cells function exerted by fasentin is most likely independent of a modulation of glucose metabolism. Taken together, our results show a novel biological activity of fasentin, which could be evaluated for its utility in cancer and other angiogenesis-dependent diseases.

## Introduction

Angiogenesis, the formation of new blood vessels from pre-existing ones, is a physiological and transient process that in adults is limited to some processes related to reproductive cycles, wound healing and bone repair. Nevertheless, a persistent and deregulated angiogenesis takes place in cancer and other diseases, such as proliferative retinopathies, psoriasis, rheumatoid arthritis and diabetes, among others^1^. As a matter of fact, angiogenesis is considered one of the so-called hallmarks of cancer^2^. Furthermore, Judah Folkman suggested in 1971 that inhibition of angiogenesis could be a revolutionary therapy against tumour growth^3^. Many factors regulate the angiogenic process, including vascular endothelial growth factor (VEGF), which acts through its receptor VEGFR2, and the fibroblast growth factor (FGF) family, such as FGF-2^4^.

**Figure 1:**
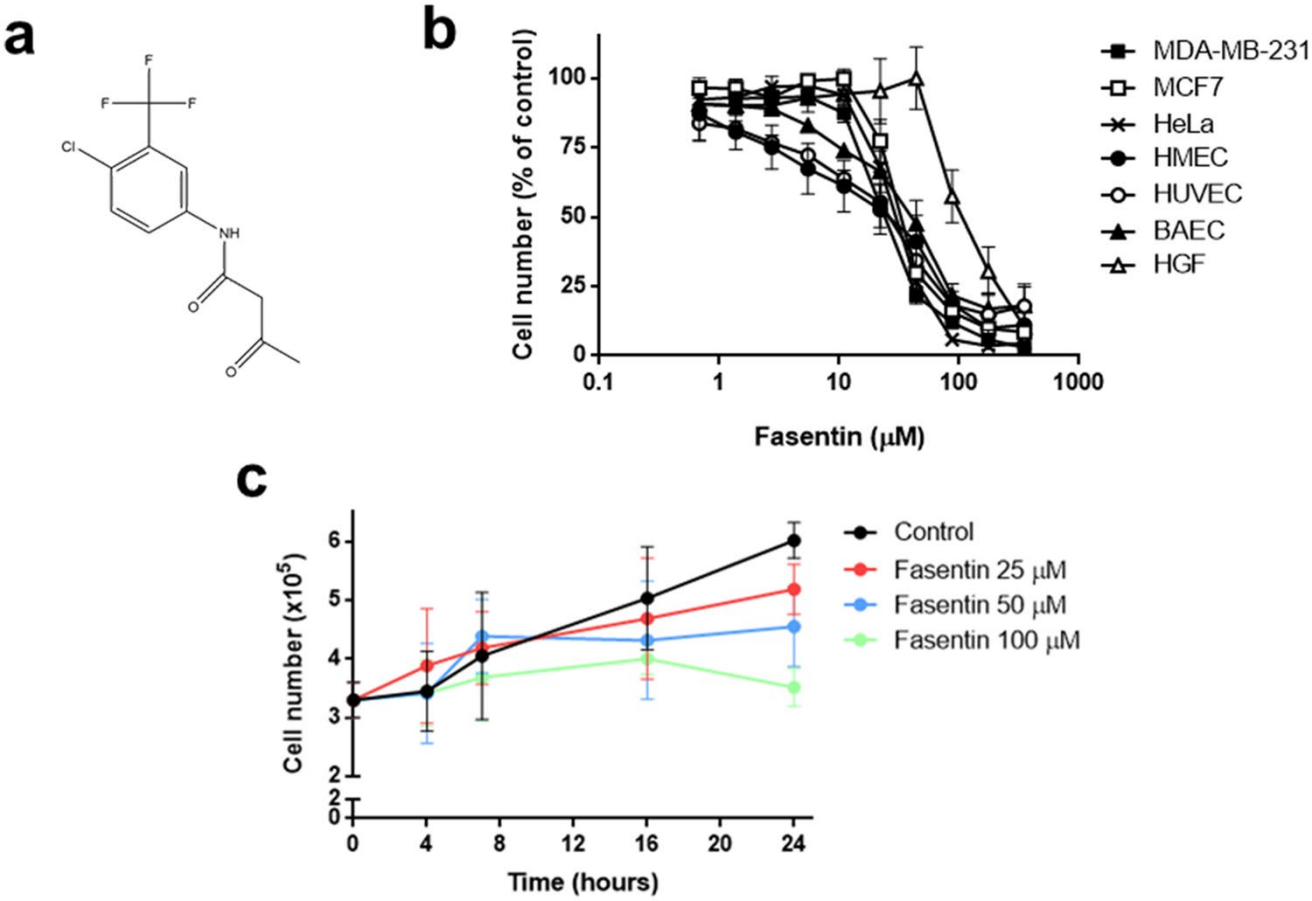
Fasentin inhibits endothelial, tumour and fibroblast cell growth. (**a**) Chemical structure of fasentin. (**b**) Dose-response curves showing the effect of fasentin on the *in vitro* growth of different cell lines after 72 h treatment from low density seeding conditions. Cell number is represented as the percentage of cells compared to the condition containing no drug. Concentrations are represented in logarithmic scale. (**c**) HMECs were seeded at high density and allowed to attach for 24 h. Then, different concentrations of fasentin were added and cell number was monitored using Trypan blue and a Neubauer chamber at 0, 4, 7, 16 and 24 h. Trypan blue allowed to exclude dead cells. Data are expressed as means ± SD of three independent experiments.

The small-molecule N-[4-chloro-3-(trifluoromethyl)phenyl]-3-oxobutanamide (fasentin, Fig. 1a) is a chemical sensitizer to the death receptor stimuli FAS, due to its effect on glucose uptake. Wood *et al*. demonstrated that fasentin at a concentration of 80 µM halved glucose uptake in leukaemia and prostate cancer cells. A virtual model marked GLUT-1 and GLUT-4 as the targets for the action of this compound^5^. Treatment with fasentin allowed the identification of the potential role of GLUT-1 in glucose uptake in corticotropinomas^6^. These results may lead to anti-tumour therapies by targeting glucose metabolism. Interestingly, silibinin, a GLUT-4 transporter inhibitor, has been seen to inhibit angiogenesis^7,8^. Additionally, several compounds that affect different steps of glucose metabolism have also been shown to prevent the angiogenesis triggering^9–12^.

Interestingly, reprogramming of energy metabolism is also considered a hallmark of cancer^13^. Recently, it has been found that regulation of some pro-angiogenic molecules is linked to glucose metabolism of endothelial cells (ECs). De Bock *et al*. observed that inhibition of glycolysis by blockade of the enzyme 6-phosphofructo-2-kinase/fructose-2,6-biphosphatase 3 (PFKFB-3) disrupted the angiogenic process^14^. It has been seen that the final product of glycolysis, lactate, has a major role in all this through modulation of IL-8, VEGF, VEGFR2 and FGF-2 expression, among others^15–18^. All in all, glucose metabolism seems to be essential for EC function. This fact opens new horizons to treat angiogenesis under pathological conditions through a metabolic approximation instead of just targeting pro-angiogenic molecules^19,20^.

In this work, we studied the possible anti-angiogenic capacity of fasentin. Our results point out to two main conclusions: 1) Fasentin is a modest inhibitor of EC proliferation, invasion and tube formation; and 2) the observed angiogenic modulatory capacity of fasentin is most likely independent of the inhibition of GLUT-1.

## Results

### Fasentin inhibits growth of endothelial cells without compromising their survival

Angiogenesis involves local proliferation of ECs. In order to test whether fasentin inhibits the proliferation of actively growing cells, we performed the MTT assay. Cells were seeded at low density and treated with different concentrations of fasentin for 72 h. Half-maximal inhibitory concentration values (IC_50_) for different cell lines are collected in Table 1. Figure 1b shows typical cell number curves for the cells tested treated with fasentin at different doses. Tested cells included three types of ECs (HMEC, human microvascular endothelial cells; HUVEC, human umbilical vein endothelial cells; and BAEC, bovine aortic endothelial cells), three human tumour cell lines (MDA-MB-231 and MCF7 breast carcinoma cells, and HeLa cervix adenocarcinoma cells), and human gingival fibroblasts (HGF). The results show that fasentin reduces the number of not only ECs, but also of tumour cells and, to a lesser extent, fibroblasts, not having a specific effect.

**Table 1:** Half-maximal inhibitory concentration IC_50_ values (μM) for fasentin treatment in different cell lines determined by the MTT assay. Data are expressed as means ± SD of three independent experiments with quadruplicate samples each.

	IC_50_ (μM)
MDA-MB-231	26.3 ± 4.8
MCF7	34.7 ± 4.0
HeLa	31.9 ± 1.4
HMEC	27.9 ± 14.5
HUVEC	27.6 ± 3.7
BAEC	42.7 ± 9.5
HGF	111.2 ± 27.0

**Figure 2:**
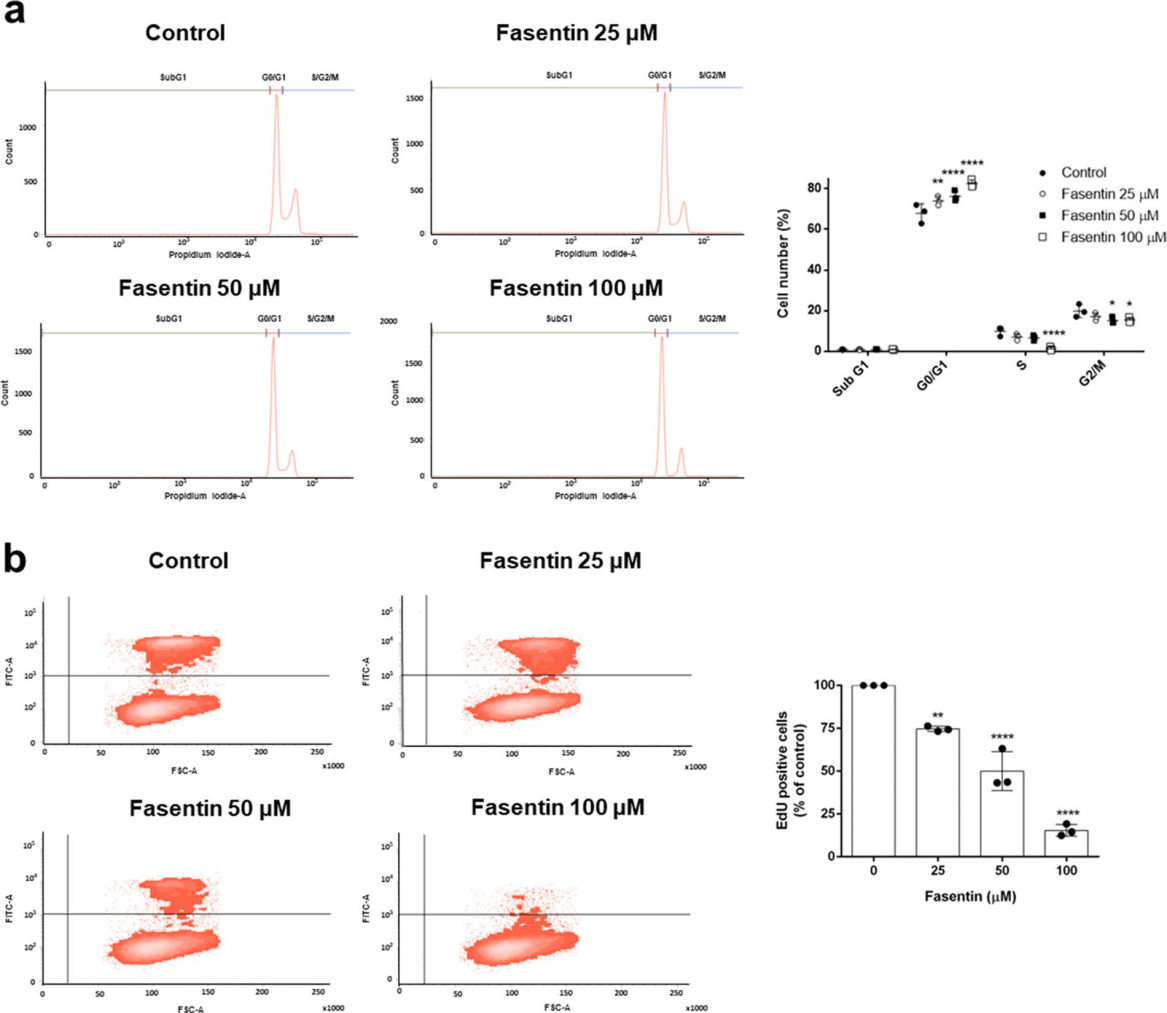
Effect of fasentin on endothelial cell cycle distribution. (**a**) HMECs were exposed for 24 h to fasentin at the indicated concentrations, stained with propidium iodide and percentages of cells on subG1, G1, S and G2/M phases were determined using a FACS VERSE^TM^ cytometer. (**b**) 10 μM EdU was added for 2 h to cells treated with fasentin for 22 h and EdU incorporation was determined using a FACS VERSE^TM^ cytometer. A representative result and quantification, expressed as means ± SD of three independent experiments, are shown. *p < 0.05, **p < 0.01, ****p < 0.0001 versus untreated control.

The IC_50_ suggests which concentrations are safe to be used for additional experiments. However, it does not give information about whether the reduction in cell number is due to an inhibition of proliferation or to an induction of cell death. For that reason, we performed additional experiments in order to clarify how fasentin reduces cell number in ECs. First, we seeded HMECs and once they reached sub-confluence we added different concentrations of fasentin. Cell number was monitored using a Neubauer chamber at 0, 4, 7, 16 and 24 h. Figure 1c shows that fasentin inhibits the increase in cell number in a dose-dependent manner, without reducing the cell number below the initial one (0 h). This suggests that fasentin does not induce cell death but it inhibits cell proliferation. To confirm this assessment, cell cycle analysis was performed in HMECs using propidium iodide staining. Flow cytometry analysis showed that fasentin does not increase the subG1 population, an indicator of apoptosis, in HMECs after either 16 h (Fig. S1) or 24 h (Fig. 2a). Instead, this compound induced a cell cycle arrest in G0/G1 phase and reduced the cell number in S phase in a dose-dependent manner (p < 0.05) (Figs. 2a and S1). These results support that fasentin inhibits cell proliferation without compromising cell survival. A more sensitive proliferation assay, such as the EdU proliferation assay, was used in order to confirm this effect on cell proliferation. Accordingly, the results obtained verified that fasentin diminishes HMECs proliferation in a dose-dependent manner (Fig. 2b).

### Fasentin inhibits capillary tube formation by endothelial cells on Matrigel

**Figure 3:**
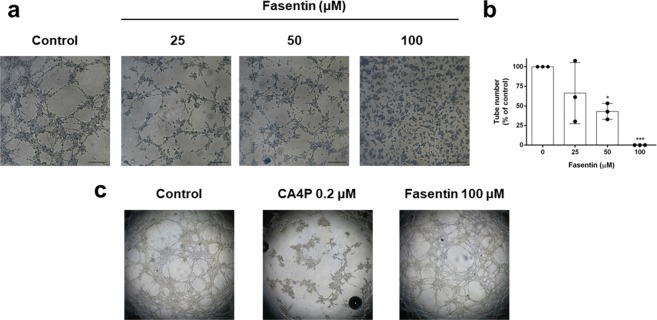
Fasentin inhibits endothelial cell tube formation *in vitro*. (**a**) Representative photographs of control (untreated) and fasentin-treated HMECs on tube formation on Matrigel. Control cells formed tubes (left panel). 100 μM fasentin completely inhibited HMECs alignment and cord formation, with partial inhibition observed at 25 and 50 μM. Cells were photographed 5 h after seeding and drug administration under an inverted microscope (bar = 200 μm). (**b**) Quantitative analysis of “tubular” structures formed. (**c**) Representative photographs of control (untreated) and fasentin-treated HMECs in the “tubule-like” structure disruption assay (bar = 500 μm). CA4P was used as positive control. Data are represented as means ± SD for three independent experiments, with duplicate samples each. *p < 0.05, ***p < 0.001 versus untreated control.

In the final event of angiogenesis, ECs form a three-dimensional network of new tubes. We can simulate this event seeding ECs on Matrigel and observing the formation of tube-like cords. As seen in representative images and quantification of tube number in Fig. 3a,b, 25 and 50 µM fasentin partially inhibited tube formation in HMECs, whereas a concentration of 100 µM exerted a total inhibition of *in vitro* tube formation. The addition of fasentin after the formation of “tubule-like” structures on Matrigel did not produce a disruption of these tubes (Fig. 3c). Therefore, fasentin is able to inhibit tube formation *in vitro* but it does not behave as a vascular disruption agent in this model.

### Fasentin impairs in vivo angiogenesis

**Figure 4:**
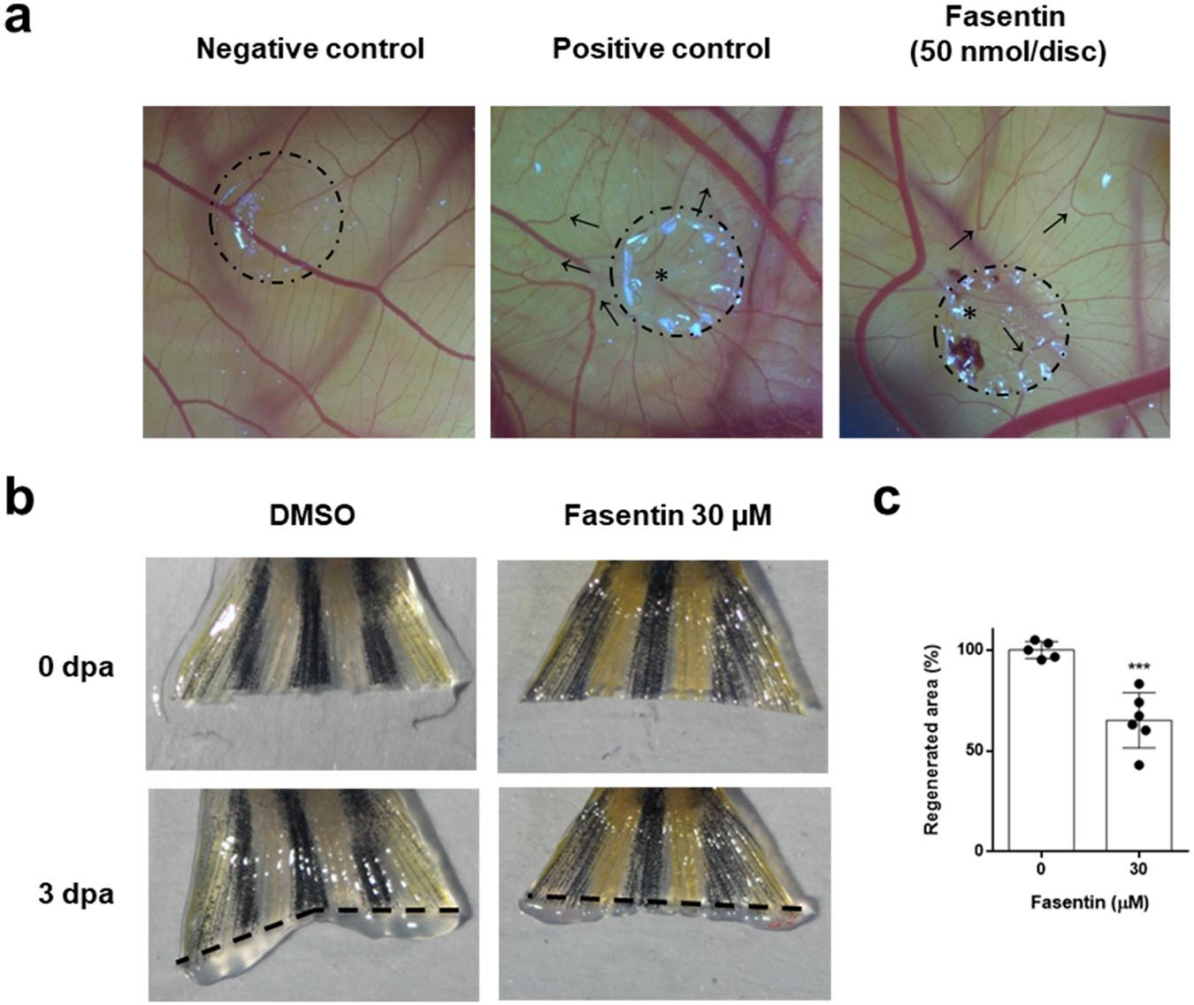
Fasentin impairs *in vivo* angiogenesis. (**a**) Chorioallantoic membrane (CAM) assay with fasentin. Methylcellulose discs containing the substance vehicle alone (DMSO) (left panel), 3 nmol aeroplysinin-1 as positive control (central panel) and 50 nmol fasentin (right panel) were added to the CAMs. Circles show the locations of the methylcellulose discs. Arrows point to rebound of vessels outward from the treated area. Asterisks indicate disrupted vessels. Additional photographs are collected in the supplementary information. (**b**) Representative photographs of caudal fin regeneration assay in WT adult zebrafish in control condition (DMSO) or treated with 30 µM fasentin after 3 dpa (days post amputation). (**c**) Quantitative analysis of the regenerated area of the caudal fin after fasentin treatment. Data are represented as means ± SD of n = 5 for control condition and n = 6 for fasentin condition. ***p < 0.001 versus untreated control.

The chick chorioallantoic membrane (CAM) assay was used to determine the ability of fasentin to inhibit angiogenesis *in ovo*. In controls, blood vessels formed a dense and spatially oriented, branching network of vascular structures whose diameter decreases as they branch (Fig. 4a, left panel). Aeroplysinin-1 was used as positive control for the inhibition of angiogenesis in this model (Fig. 4a, central panel)^21^. Fasentin was able to impair angiogenesis *in ovo* with a dose of 50 nmol per disc, mainly observed by the apparent vessels rebounds close to the methylcellulose discs (never observed in the DMSO condition), as well as an alteration of the general pattern of vascularization in the CAM, compared to the regular and hierarchic network observed in the DMSO controls (Fig. 4a, right panel and Fig. S2). Table 2 summarizes the evaluation of the *in ovo* impairment of angiogenesis in the CAM assay by fasentin, understood as the number of eggs out of the total number of evaluated eggs in which some of these altered vascular characteristics were detected. 50 nmol fasentin was the most effective amount of this compound for the modulation of angiogenesis in this model. Lower amounts of fasentin affected angiogenesis only in a reduced percentage of the total evaluated eggs.

**Table 2:** Impairment of *in ovo* angiogenesis by fasentin. Percentage of treated egg CAMs that showed some degree of impairment of angiogenesis after fasentin treatment.

Fasentin (nmol/disc)	Positives/total	Inhibition fraction
0	0/21	0
1.25	0/4	0
2.5	0/4	0
5	2/10	0.2
12.5	4/16	0.2
25	4/18	0.2
50	7/8	0.9

Additionally, the zebrafish caudal fin regeneration assay was performed. An active angiogenesis has been found to be necessary for proper fin regeneration in this model^22^. The results show a significant decrease in the regenerated area of the caudal fin of animals treated with 30 µM fasentin compared to the control condition (p < 0.001) (Fig. 4b,c).

### Fasentin does not affect the migratory capability of endothelial cells

**Figure 5:**
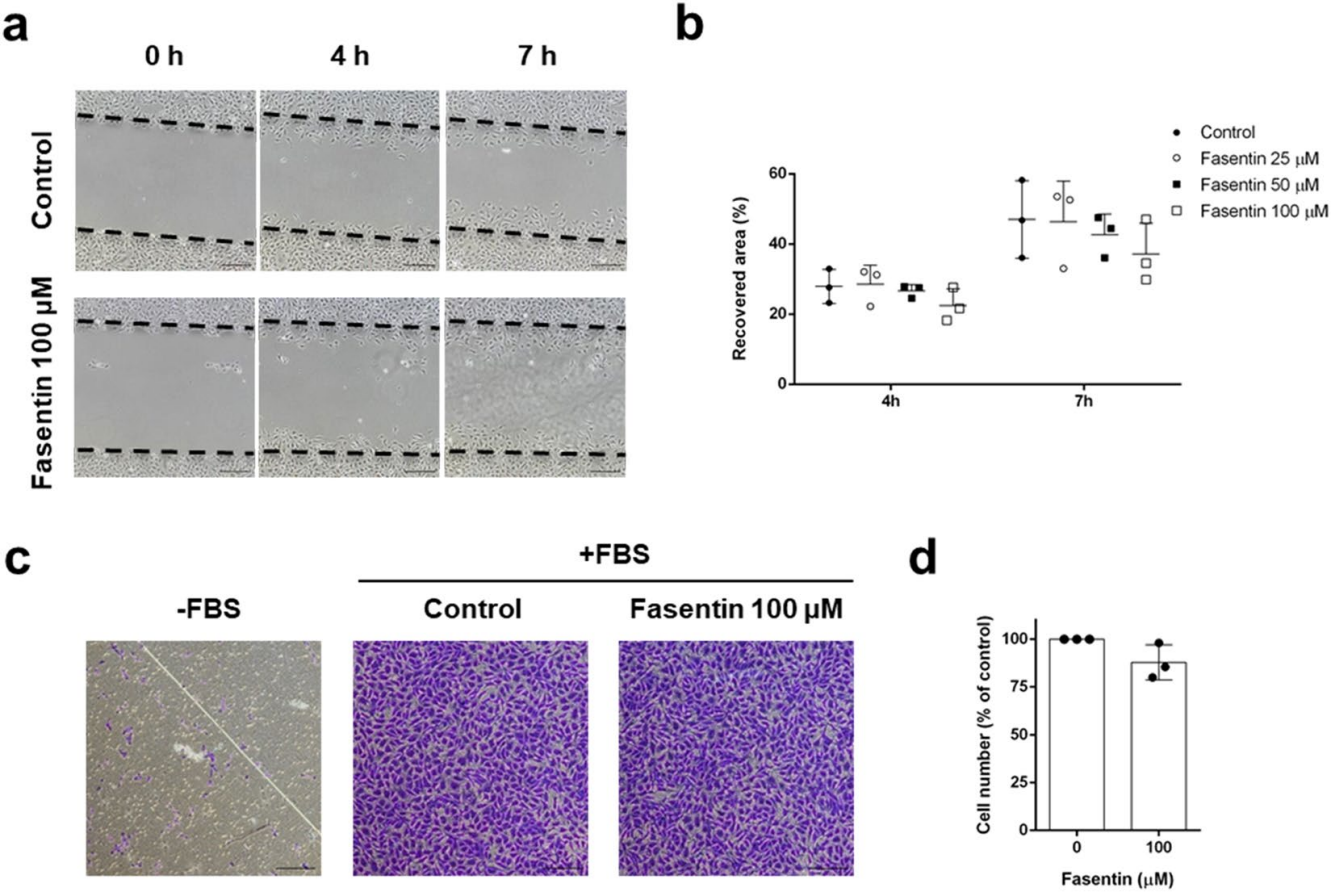
Effect of fasentin on endothelial cell migration *in vitro*. (**a**) Representative images of control and fasentin-treated HMECs at the beginning of the assay and after indicated times of incubation (bar = 200 μm). (**b**) Quantification of recovered area. (**c**) Representative photographs of control and fasentin-treated HMECs after 16 h incubation in a transwell insert coated with gelatine. FBS was added as chemoattractant to the lower wells (bar = 200 μm). (**d**) Quantification of migrated cells. Data are expressed as means ± SD of three independent experiments.

After confirming the modulatory capacity of fasentin on angiogenesis *in vitro* and *in vivo*, we tried to further characterize the stages of this process that could be affected by this compound. During the angiogenic process, ECs need to migrate in order to form a new blood vessel. We performed the “wound healing” assay to test the possible effect of fasentin on EC migration. Figure 5a,b show that fasentin does not have a significant effect on HMECs migration. The lack of effect of this compound on EC migration was confirmed by means of a Boyden chamber assay at a longer incubation time. An overnight incubation with 100 µM fasentin did not have a statistically significant effect on HMECs migration (Fig. 5c,d).

### Fasentin diminishes endothelial cell potential to remodel extracellular matrix and invade

**Figure 6:**
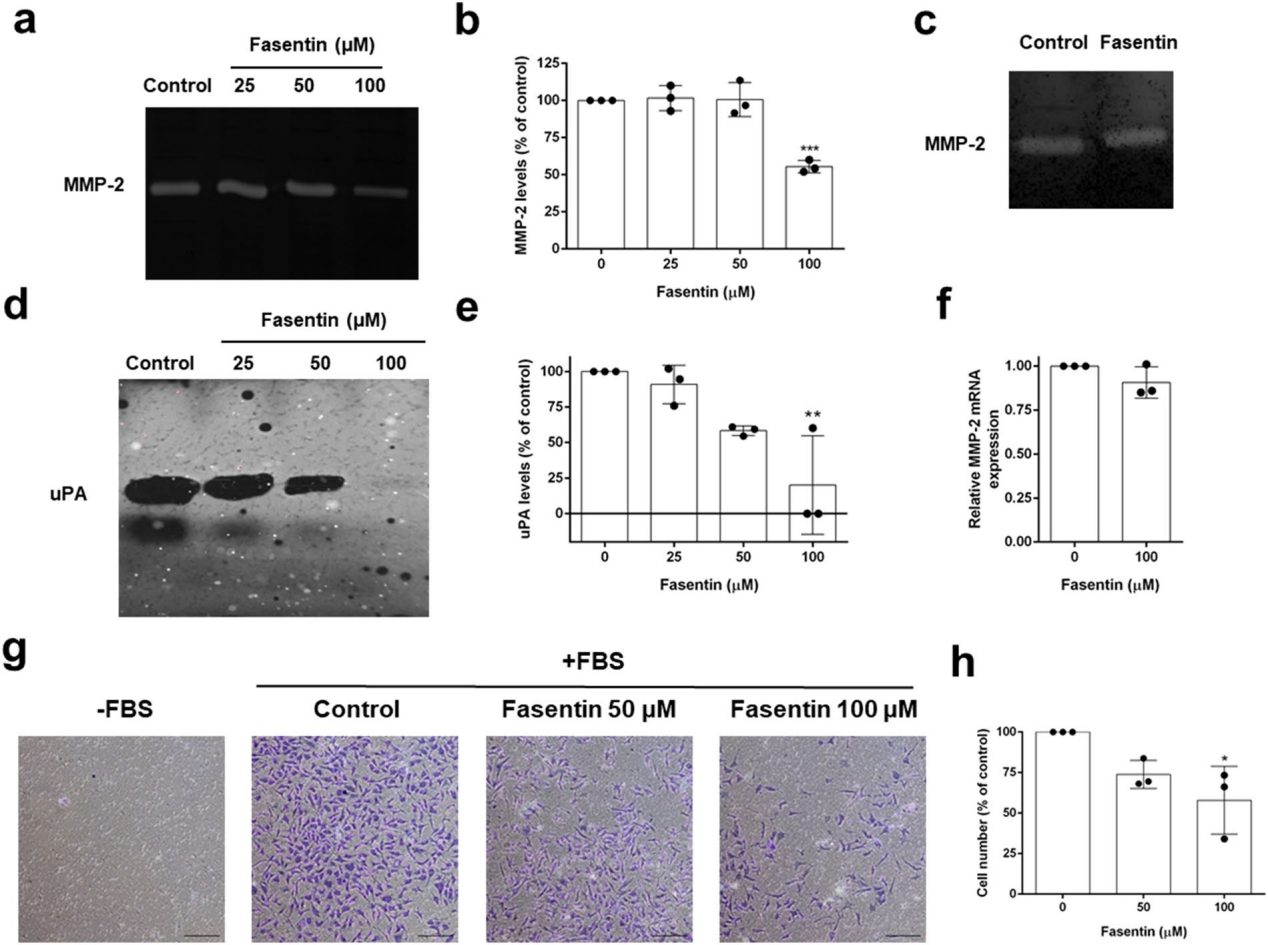
Fasentin decreases the levels of molecules related to the remodelling of the extracellular matrix and inhibits EC invasion in HMECs. (**a**) Cell extracts from HMECs treated for 16 h with the indicated concentrations of fasentin were normalized for equal cell density and used for gelatine zymography as indicated in the “Methods” section. (**b**) Quantification of the relative MMP-2 levels. (**c**) Cells extracts from the control condition were used for *in situ* gelatine zymography. 100 μM fasentin was added to cut lanes of the gel, incubated for 16 h and stained with Coomassie Brilliant Blue. (**d**) Representative image of uPA activity in cell lysates from control and fasentin-treated HMECs for 16 h. (**e**) Quantification of the relative uPA levels. (**f**) Relative MMP-2 mRNA expression after 8 h treatment with 100 μM fasentin. (**g**) Representative photographs of control and fasentin-treated HMECs after 16 h incubation in a transwell insert coated with Matrigel. FBS was added as chemoattractant to the lower wells (bar = 200 μm). (**h**) Quantification of invasive cells. Data are given as percentage of the untreated control, and expressed as means ± SD of three independent experiments. *p < 0.05, **p < 0.01, ***p < 0.001 versus untreated control. Uncropped blots are shown in the supplementary information.

ECs can produce and secrete to the media extracellular matrix (ECM) remodelling enzymes such as metalloproteinases (MMPs) and the urokinase-type plasminogen activator (uPA). These enzymes play an essential role in angiogenesis allowing the migration of ECs through the ECM surrounding them, a process commonly known as cell invasion. Gelatine and casein zymographies enable quantification of MMPs and uPA activity, respectively, in a simple experiment. Figure 6a,b show that 100 µM fasentin was able to decrease MMP-2 levels in cell extracts from HMECs (p < 0.001). No effect was found on MMP-2 levels in the culture media (Fig. S3), suggesting that the effect of fasentin involves a decrease on MMP-2 production rather than secretion. Moreover, fasentin did not affect MMP-2 activity directly, as seen by an *in situ* gelatine zymographic assay (Fig. 6c). On the other hand, fasentin inhibited uPA levels in HMECs extracts in a dose-dependent manner, with no effect at 25 µM (Fig. 6d,e). Afterwards, we tried to elucidate whether this reduction of MMP-2 and uPA production was regulated at the translational level. However, we did not detect any changes in MMP-2 mRNA expression (Fig. 6f) or in mRNA expression of the MMP inhibitors TIMPs 1–4 (data not shown). mRNA expression of uPA, uPA receptor (uPAR) and PAI-1, and inhibitor of uPA, was not detected.

Additionally, fasentin was able to inhibit cell invasion through Matrigel on a Boyden chamber assay in a dose-dependent manner (Fig. 6g,h).

### The anti-angiogenic activity of fasentin is most likely independent of glucose metabolism modulation

**Figure 7:**
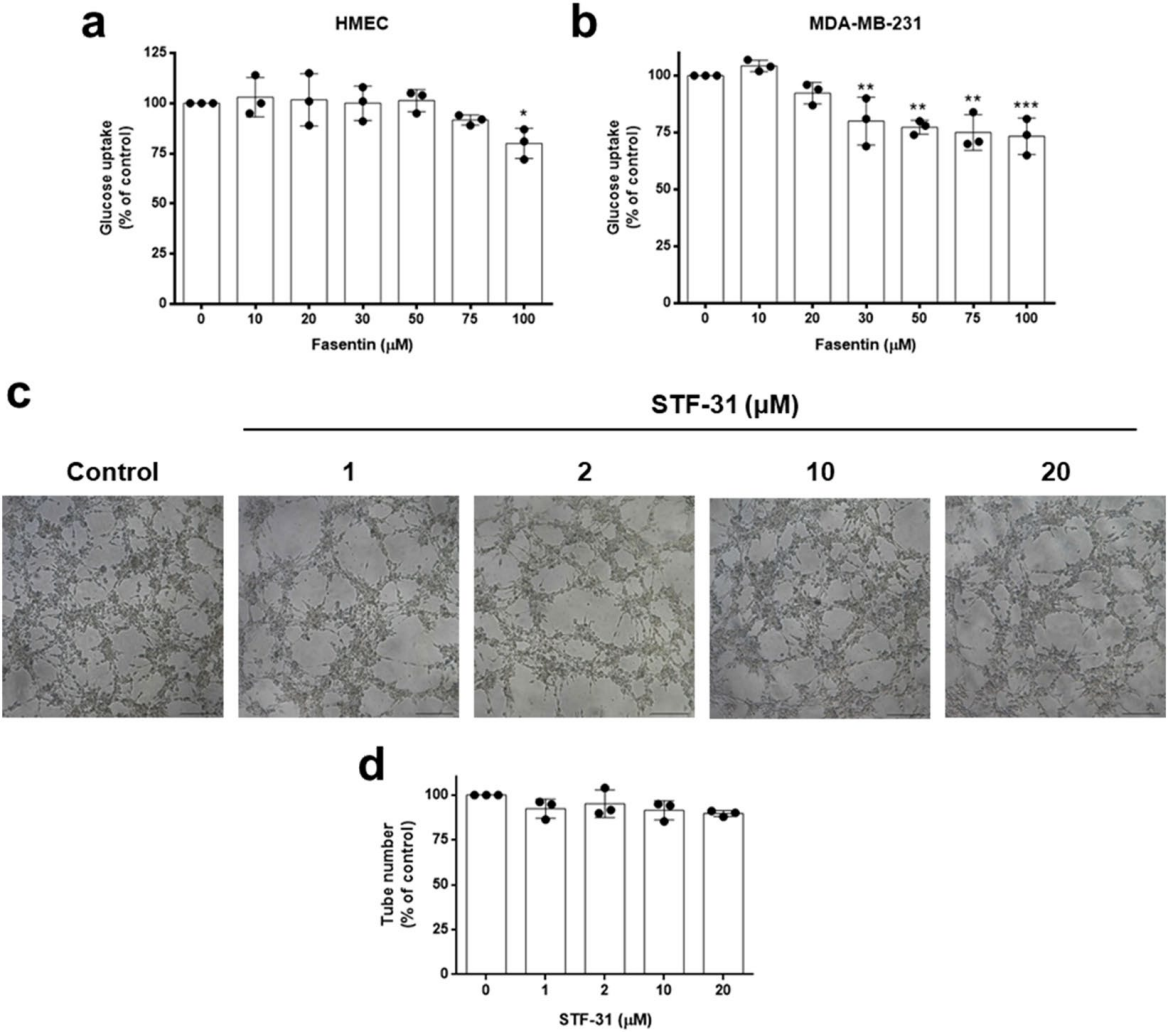
Effect of fasentin on glucose uptake in endothelial and tumour cells. (**a**) Glucose uptake was determined with FACS analysis using the glucose fluorescent analogue 2-NBDG. Fluorescent relative units are shown for HMECs and (**b**) MDA-MB-231 cells in the presence of different concentrations of fasentin. (**c**) Representative photographs of control (untreated) and STF-31-treated HMECs on tube formation on Matrigel (bar = 200 μm). (**d**) Quantitative analysis of “tubular” structures formed. Data are expressed as means ± SD of three independent experiments. *p < 0.05, **p < 0.01, ***p < 0.001 versus untreated control.

Next, we examined whether the doses that affected HMECs proliferation, invasion and tube formation also affected glucose uptake in these cells. However, only a slight effect on glucose uptake in HMECs was observed at 100 µM (p < 0.05) (Fig. 7a). Because this compound was described as a glucose uptake inhibitor in tumour cells, we also tested the role of fasentin in glucose uptake in MDA-MB-231 breast cancer cells. This compound diminished glucose uptake in these tumour cells at lower concentrations than in HMECs, exhibiting a little yet statistically significant effect at concentrations as low as 30 µM (p < 0.01) (Fig. 7b).

In order to corroborate whether a modulation of GLUT-1 activity could affect angiogenesis, we tested the possible anti-angiogenic capacity of STF-31, a specific GLUT-1 inhibitor^23^. However, STF-31 did not affect tube formation by HMECs on Matrigel (Fig. 7c,d). Therefore, an additional mechanism must be involved in the anti-angiogenic activity of fasentin.

### Fasentin does not inhibit the tyrosine kinase activity of VEGFR2

VEGFR2 is a tyrosine kinase receptor that plays a central role in angiogenesis. Upon ligand binding, VEGFR2 undergoes autophosphorylation and becomes activated. Inhibition of this receptor would result in an inhibition of VEGF-mediated angiogenesis. By means of a commercial *in vitro* VEGFR2 kinase assay kit, we tested whether fasentin inhibits VEGFR2 kinase activity. Our results show that 100 µM fasentin did not significantly decrease VEGFR2 kinase activity (the remaining kinase activity relative to the vehicle control was 83.4 ± 18.6%, expressed as mean ± SD (p = 0.3157)). 1 µM sunitinib, a well-known inhibitor of VEGFR2 tyrosine kinase activity, was used as positive control, yielding a total inhibition of the kinase activity^24,25^.

### Fasentin affects the ERK1/2 and PI3K/Akt signalling pathways

**Figure 8:**
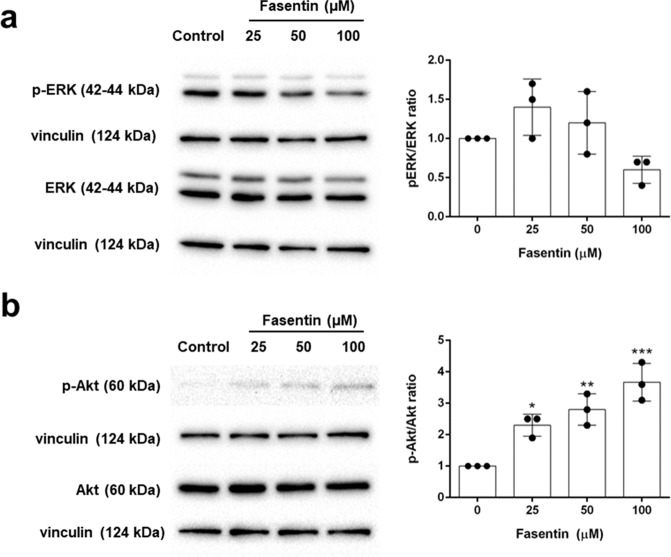
Effect of fasentin on the activation of the ERK and PI3K/Akt signalling pathways. (**a**) Representative blots and quantification of phosphorylated ERK1/2, total ERK1/2, (**b**) phosphorylated Akt and total Akt in protein extracts from HMECs treated with different concentrations of fasentin for 16 h. Data are expressed as means ± SD of three independent experiments. *p < 0.05, **p < 0.01, ***p < 0.001 versus untreated control. Uncropped blots are shown in the supplementary information.

The ERK1/2 signalling pathway is known to be involved in EC proliferation, differentiation and morphogenesis, and ECM degradation^26^. Even though the effect was not statistically significant, we found lower levels of phospho-ERK in HMECs treated with 100 µM fasentin, indicating a partial inhibition on the ERK signalling pathway (Fig. 8a).

On the other hand, activation of the PI3K/Akt signalling pathway leads to EC proliferation and survival^27^. Fasentin was found to greatly induce Akt phosphorylation in a dose-dependent manner, thus activating the PI3K/Akt signalling pathway (Fig. 8b).

## Discussion

Angiogenesis and EC metabolism have been demonstrated to be closely connected^28–31^. Glycolysis, in particular, has been seen to be essential for correct EC function^14^. Thus, targeting glucose metabolism in these cells could be a tool for inhibiting angiogenesis^19^. Fasentin is a synthetic GLUT-1 and GLUT-4 inhibitor able to diminish glucose uptake in several cancer cell lines^5,32–34^. Since other glucose metabolism inhibitors have also been characterized as anti-angiogenic compounds, we decided to test whether fasentin could be an angiogenic modulator^7,35–38^. For this aim, we mainly used human dermal microvascular ECs. These HMECs are a healthy EC line that could behave differently than a pathogenic EC line such as tumour ECs. In the experiments carried out in this work, we seeded the cells so that when we added fasentin the cells were still growing, simulating their proliferating, “pathogenic” phenotype. This experimental approach may be useful for a preliminary study of the anti-angiogenic capacity of a compound. Additional experiments using tumour ECs, for instance, could be performed in order to deepen in the specific effects/mechanism of that compound, including whether that compound affects the metabolic shift of ECs in that model. This fact should be taken into account in order to interpret the results herein shown.

Our data show that fasentin inhibits EC proliferation without compromising cell survival (Figs. 1 and 2). In some cancer cells, but not all, fasentin is able to produce cell death at some extent^5,32^. However, fasentin did not induce cell death in HMECs, but it decreased cell proliferation, as seen by the results from the cell growth curves and the cell cycle analysis. This cell cycle analysis also showed that 100 µM fasentin induced a cell cycle arrest in G0/G1 phase and decreased the number of cells in S phase (Figs. 2a and S1), corroborating the effect on cell proliferation. This fact was also observed with fasentin in leukaemia cells^5^. The same effect was seen with other GLUT inhibitors such as silibinin and naringenin^7,35^. Additionally, EdU incorporation was lower in HMECs treated with fasentin in a dose-dependent manner (Fig. 2b), confirming the decrease in cell proliferation exerted by fasentin. Therefore, all these results suggest that fasentin has not a cytotoxic effect in HMECs at the concentrations tested, but it has a cytostatic effect in a dose-dependent manner.

After corroborating its lack of cytotoxicity, we used 25, 50 and 100 µM fasentin in order to test its potential effect on angiogenesis *in vitro*. Our results demonstrate for the first time that fasentin inhibits in a dose-dependent manner certain functions of ECs, namely proliferation, differentiation and invasion. The anti-angiogenic activity of fasentin was first detected using the *in vitro* tube formation assay in ECs. 100 µM fasentin was able to completely inhibit tube formation in HMECs, while 50 µM fasentin had a partial effect (Fig. 3a,b). The highest tested dose of fasentin did not disrupt pre-formed tubes, as seen in the “tubule-like” structure disruption assay (Fig. 3c). On the other hand, a disorganized, altered patter of vascularization in the CAM of chick eggs was found at 50 nmol/disc of fasentin (Fig. 4a and Table 2). Moreover, a significant effect was also found in the zebrafish caudal fin regeneration assay (Fig. 4b,c). Therefore, fasentin is also able to impair neovascularization *in vivo*.

There are different steps in the angiogenic process that can be inhibited by anti-angiogenic compounds. Migration of ECs is necessary for angiogenesis. Several anti-angiogenic drugs have been shown to inhibit migration of ECs^39–43^, while others inhibited angiogenesis without having a great effect on migration^44,45^. Our data show that fasentin does not have a significant effect on EC migration, demonstrated by means of two different experimental approximations (Fig. 5). However, in order to migrate, ECs firstly need to degrade the surrounding ECM. Therefore, the remodelling of the ECM is another requirement for the angiogenic process. MMPs are the enzymes responsible for the degradation of type IV collagen and hence they are needed to trigger angiogenesis. ECs are known to express MMP-2^46–48^. We could not detect an effect of fasentin on MMP-2 secretion to the media (Fig. S3), but a decrease in MMP-2 levels in cell extracts from cells treated with 100 µM fasentin was found (Fig. 6a,b). This effect was not accompanied by either a decrease in the activity of the gelatinase or in its mRNA expression (Fig. 6c,f). Since we did not detect an effect of fasentin on MMP-2 levels in the media but we did in the cell extracts, it is most likely that MMP-2 production rather than secretion is affected by this compound. Due to this inhibition of MMP-2 production, a decrease in secreted MMP-2 levels may be found after longer incubation times. Despite the low effect of fasentin on MMP-2 levels, 50 and 100 µM fasentin clearly diminished EC invasion (Fig. 6g,h). These results suggest that additional molecules may be involved in the inhibition of EC invasion exerted by fasentin. Urokinase is another proteinase involved in ECM remodelling and associated with the angiogenic process^49^. Fasentin greatly affected uPA levels in HMECs (Fig. 6d,e). Whether this decrease in uPA levels is sufficient for the inhibition of EC invasion exerted by fasentin or if additional mechanisms are involved should be further researched.

In order to test the possible correlation between the anti-angiogenic capacity of fasentin and its effect on GLUT transporters, we studied the effect of the concentrations of fasentin used for the characterization of the anti-angiogenic activity of this compound on the glucose uptake in ECs. However, our results show that 100 µM fasentin only slightly diminished glucose uptake in HMECs, whereas concentrations bellow 75 µM did not have any effect on glucose uptake (Fig. 7a). Remarkably, in an independent work we found out that HMECs express two different glucose transporters, namely GLUT-1 and GLUT-14 (data not published). Thus, it is likely that HMECs rely on GLUT-14 for glucose uptake when GLUT-1 activity is compromised. In addition, it is described that fasentin inhibits GLUT-1, yet just partially^5^. Therefore, these facts could explain the slight effect of fasentin on glucose uptake in HMECs. Moreover, we tested whether a specific GLUT-1 inhibitor, such as STF-31, could inhibit tube formation in these cells. However, contrary to fasentin, no effect was found for STF-31 on tube formation (Fig. 7c,d). All in all, the role of fasentin in EC proliferation, differentiation and invasion is most likely independent of the inhibition of GLUT-1 transporters and no correlation between these apparently independent effects should be made.

Therefore, we tried to elucidate the mechanism by which fasentin inhibits proliferation, invasion and tube formation in ECs. VEGFR2 is a tyrosine kinase receptor that plays a central role in angiogenesis. Upon ligand binding, VEGFR2 undergoes autophosphorylation and becomes activated. However, no significant effect of 100 µM fasentin was found on the tyrosine kinase activity of VEGFR2.

On the other hand, the angiogenesis triggering is regulated by a complex network of signalling pathways, which finally controls the response of ECs to pro-angiogenic stimulus. The ERK1/2 signalling pathway is known to be involved in EC proliferation, differentiation and morphogenesis, and ECM degradation^26^. Although it was not statistically significant, we found a slight decrease in the phosphorylation of ERK1/2 in HMECs treated with 100 µM fasentin, indicating a partial inhibition of the ERK signalling pathway (Fig. 8a). However, since this decrease was rather small and lower doses of fasentin also affected cell proliferation and invasion, but did not inhibit the ERK1/2 signalling pathway, additional mechanisms are most likely involved in the modulatory activity of fasentin in several features of ECs. The PI3K/Akt signalling pathway is also involved in the angiogenic function. Activation of Akt leads to EC proliferation and survival^27^. Our results show that fasentin increased the phosphorylated form of Akt in a dose-dependent way (Fig. 8b). Inhibition of cell proliferation usually leads to cell death. However, fasentin decreases cell proliferation without compromising cell survival. This could be explained due to the increased activation of the PI3K/Akt signalling pathway with this treatment. Moreover, this pathway is also known to be involved in the regulation of glucose metabolism. Among other effects, activation of Akt leads to an increase of GLUT-1 expression^50,51^. Therefore, it could be possible that the inhibition of GLUT-1 by fasentin leads to an activation of Akt in order to compensate for the lower glucose transport. As a consequence, more molecules of GLUT-1 would be available in the plasma membrane. This, along with the presence of GLUT-14 in HMECs, could explain the low effect of fasentin on glucose uptake in these cells. However, due to the lack of anti-angiogenic capacity of the GLUT-1 inhibitor STF-31 in these cells, modulation of GLUT-1 activity is not likely involved in the effect of fasentin on EC proliferation, differentiation and invasion. Further research should be performed in order to elucidate the exact mechanism of fasentin in EC function.

In summary, we have shown that fasentin inhibits tube formation *in vitro* probably through inhibition of EC proliferation and invasion. This effect seems to be just partially due to a decrease in MMP-2 and uPA production and to a modulation of the ERK1/2 signalling pathway. Moreover, fasentin barely inhibits glucose uptake in these ECs and another GLUT-1 inhibitor did not affect tube formation in these cells. Therefore, the effects found for fasentin on EC proliferation, differentiation and invasion in this work are most likely independent of a modulation of glucose metabolism.

## Methods

### Materials

MCDB-131 cell culture medium was obtained from Gibco (Paisley, Scotland, UK). Other cell culture media, penicillin, streptomycin and amphotericin B, and trypsin were purchased from BioWhittaker (Verviers, Belgium). Fetal bovine serum (FBS) was purchased from Biowest (Kansas, USA). Matrigel was purchased from Becton-Dickinson (Bedford, MA, USA). Fasentin was supplied by Calbiochem (San Diego, CA, USA), dissolved in DMSO as a 70 mM stock and stored at −20 °C. In all the assays, the vehicle (DMSO) was lower than 0.14% (v/v). STF-31 was supplied by Sigma-Aldrich (St. Louis, MO, USA), dissolved in DMSO as a 40 mM stock and stored at −20 °C. 2-NBDG was supplied by Molecular Probes (Eugene, OR, USA). All the antibodies used in this work were from Cell Signaling Technology (Danvers, MA, USA). Fertilized chick eggs were obtained from Granja Santa Isabel (Córdoba, Spain). Wild-type AB zebrafish were kindly supplied by Dr. Manuel Marí Beffa (Universidad de Málaga, Spain). Plastic material for cell culture was from Nunc (Roskilde, Denmark). All other reagents not listed on this section were from Sigma-Aldrich (St. Louis, MO, USA).

### Cell culture

All cell culture media were supplemented with glutamine (2 mM), penicillin (50 U/mL), streptomycin (50 U/mL) and amphotericin (1.25 μg/mL). Human microvascular endothelial cells (HMEC) were kindly supplied by Dr. Arjan W. Griffioen (Maastricht University, Netherlands) and maintained in MCDB-131 medium supplemented with 10% FBS, hydrocortisone (1 μg/mL) and epithelial growth factor (EGF) (10 ng/mL). Human umbilical vein endothelial cells (HUVEC) were isolated by a modified collagenase treatment, as previously reported^52^ and maintained in 199 medium supplemented with 20% FBS, endothelial cell growth supplement (ECGS) (30 µg/mL) and heparin (100 µg/mL). Bovine aortic endothelial cells (BAEC) were isolated from bovine aortic arches as previously described^44^ and maintained in Dulbecco’s modified Eagle’s medium (DMEM) containing glucose (1 g/L) and supplemented with 10% FBS. Primary human gingival fibroblasts (HGF) were maintained in DMEM containing glucose (4.5 g/L) and supplemented with 10% FBS. Tumour cells used in this paper (human breast carcinoma MDA-MB-231 and MCF7, and human cervix adenocarcinoma HeLa) were purchased from the ATCC (Rockville, MD, USA) and maintained in RPMI-1640, DMEM containing glucose (4.5 g/L) and EMEM, respectively, all supplemented with 10% FBS. All cell lines were maintained at 37 °C under a humidified 5% CO_2_ atmosphere.

### MTT assay

The 3-(4,5-dimethylthiazol-2-yl)-2,5-diphenyltetrazolium bromide (MTT) dye reduction assay in 96-well microplates was performed as previously described^21^. 2.5 × 10^3^ cells for HMEC, BAEC, MCF7 and HGF, 4 ×10^3^ cells for HUVEC, and 2 × 10^3^ cells for MDA-MB-231 and HeLa in a total volume of 100 μL of complete medium were incubated for 3 days with serial dilutions of fasentin (37 °C, 5% CO_2_ in a humidified atmosphere). Absorbance was read at 550 nm with an Eon Microplate Spectrophotometer from Bio-Tek Instruments (Winooski, VT, USA). Data were collected by Gen5 software from the same manufacturers. Four samples for every tested concentration were included in each of three independent experiments. Half-maximal inhibitory concentration (IC_50_) values were calculated as the concentrations of compound yielding 50% cell number, taking the values obtained for the control (untreated) condition as 100%.

### Cell growth curves

HMECs were seeded and once they reached sub-confluence they were treated or not with 25, 50 or 100 µM fasentin. Cell number was monitored at 0, 4, 7, 16 and 24 h after treatment using a Neubauer chamber. Dead cells were excluded by using Trypan blue.

### Cell cycle analysis assay

HMECs were seeded and treated or not with 25, 50 or 100 µM fasentin for 16 or 24 h when they were in exponential growth phase. Attached and unattached control and fasentin-treated cells were harvested, washed with PBS, and fixed with 70% ethanol for 1 h on ice. Pelleted cells were incubated with 0.1 mg/mL RNase-A and 40 µg/mL propidium iodide at 37 °C for 30 min protected from light. Percentages of subG1, G0/G1, S and G2/M populations were determined using a FACS VERSE^TM^ cytometer from BD Biosciences (San Jose, CA, USA) and data were analysed with its software BD FACSuite.

### EdU proliferation assay

HMECs were seeded and treated or not with 25, 50 or 100 µM fasentin when they were in exponential growth phase. After 22 h, 10 µM EdU was added to each well for additional 2 h. Cells were harvested and EdU incorporation was detected using a baseclick EdU Flow Cytometry Kit (Baseclick GmbH) in a FACS VERSE^TM^ cytometer from BD Biosciences (San Jose, CA, USA) and data were analysed with its software BD FACSuite.

### Tube formation and tube disruption on Matrigel by endothelial cells

Matrigel (50 µL of about 10.5 mg/mL) at 4 °C was used to coat each well of a 96-well plate and allowed to polymerize at 37 °C for a minimum of 30 min. 7 ×10^4^ cells were added with 200 µL of medium without serum. Different amounts of compound were added to the wells and incubated at 37 °C. After 5 h incubation, cultures were observed and photographed with a microscope camera Nikon DS-Ri2 coupled to a Nikon Eclipse Ti microscope from Nikon (Tokyo, Japan). Closed “tubular” structures were counted using ImageJ software.

For tube disruption assay, cells were seeded on Matrigel and fasentin was added to the wells once the “tubular” structures were formed. Cultures were observed and photographed after 90 min incubation. 0.2 µM combretastatin A-4 phosphate (CA4P) was used as positive control^53^.

### In ovo angiogenesis CAM assay

Fertilized chick eggs were incubated horizontally at 38 °C in a humidified incubator, windowed by day 3 of incubation and processed by day 8 according to developmental age of the chick due to shipping of eggs^54^. Different amounts of fasentin were added to a 1.2% solution of methylcellulose in water, and 10 µL drops of this solution were allowed to dry on a Teflon-coated surface in a laminar flow hood. These methylcellulose discs were implanted on the chorioallantoic membrane (CAM), the eggs were sealed with adhesive tape and incubated at 38 °C for additional 48 h. Negative controls were always made with DMSO mixed with the methylcellulose. Aeroplysinin-1 (3 nmol/CAM) was used as a routine positive assay control^21^. After the re-incubation, the CAM was examined under a stereomicroscope. The assay was scored as positive when two independent observers reported a significant reduction of vessels in the treated area within an egg. The number of positive eggs was then divided by the total of evaluated eggs in order to obtain the fraction of positive eggs.

### Zebrafish caudal fin regeneration assay

Adult wild-type AB zebrafish were anesthetized with 0.2 mg/mL tricaine and the caudal fin was partially amputated with a scalpel. The animals were incubated in fish water containing the vehicle control (DMSO) or fasentin at 28.5 °C for 3 days. Photographs of the caudal fin were taken at 0 and 3 dpa (days post amputation) with a Nikon DS-Ri2 camera coupled to a Nikon SMZ 745 T stereomicroscope from Nikon (Tokyo, Japan). The interpretation of the results was based on the regenerated area of the caudal fin compared to the control condition.

### Animals and ethical statement

All experimental procedures with animals were conducted in accordance with the Spanish Legislation (Real Decreto 53/2013, BOE, 34/-11421, 2013) in compliance with the European Community Directive 2010/63/EU regulating the use and care of laboratory animals. The protocols were approved by the Ethics Committee for Animal Experiments of the University of Málaga.

### Endothelial cell migration and invasion assays

The migratory capacity of HMECs was assessed using the so-called “wound-healing” assay as previously described^42^. Wounded areas were observed under microscope after 4 and 7 h incubation, and photographs were taken from the same areas as those recorded at zero time. Images were analysed with ImageJ software. The regrowth of HMECs into the cell-free area was calculated as the percentage of the initial wounded area (time 0) that had been recovered by ECs after different incubation times.

Alternatively, migration of HMECs after 16 h incubation was assayed by using a 24-well clear membrane insert. HMECs were serum-starved in medium containing 0.1% BSA for 24 h. Inserts were coated with 0.5% gelatine for 16 h at 4 °C. Then, 3 ×10^4^ cells were added to the inserts in serum-free medium in the absence or presence of fasentin. MCDB-131 with 10% FBS was used as chemoattractant in the lower wells. FBS-free medium was used as negative control. The inserts were incubated at 37 °C for 16 h, and then fixed with 4% paraformaldehyde (PFA) for 15 min at RT, washed with PBS and dyed with 1% violet crystal (on 2% ethanol) for 20 min. Inserts were washed with distilled water, and photographs were taken after the inserts were dried. Migrated cells were counted with ImageJ software and referred to control cells.

For invasion assays, the same procedure as migration was followed, but 12 µg/100 µL of Matrigel was used to coat the inserts instead of gelatine. Inserts with Matrigel were left on a flow hood overnight before seeding the cells.

### Zymographic assays for the detection of extracellular matrix remodelling enzymes activity

Cells were incubated for 16 h with media containing 0.1% BSA and 200 KIU/mL aprotinin in the absence (controls) or presence of fasentin. After the incubation, cell extracts were collected, centrifuged at 1000 x g at 4 °C for 10 min, and used for gelatine and plasminogen zymographies as previously described^21^. Duplicates were used to determine cell number with a Coulter counter. For *in situ* gelatinograms, control samples were subjected to electrophoresis. Gels were cut and the different lanes were incubated separately with the substrate buffer in the absence or presence of 100 µM fasentin for 16 h at 37 °C.

Quantitative analysis of zymographies was performed using ImageJ software and cropped blots were obtained using Adobe Photoshop CS6 software.

### RNA isolation and purification and cDNA synthesis

Cells at 80% confluence in 6-well plates were treated with or without 100 µM fasentin for 8 or 16 h. After incubation, cells were harvested and total RNA was isolated with the Direct-zol™ RNA MiniPrep Kit (Zymo Research) according to the purchaser’s instructions.

Complementary DNA (cDNA) synthesis was carried out with the High-Capacity cDNA Reverse Transcription Kit (Applied Biosystems).

### qPCR

For quantitative RT-PCR (qPCR), total RNA isolation and cDNA synthesis were performed as described above and PCR reactions were done using KAPA SYBR Fast Master Mix (2×) Universal (KAPA Biosystems) in an Eco Real-Time PCR System. The following thermal cycling profile was used: 95 °C, 3 min; 40 cycles of 95 °C, 10 s; Tm, 30 s. qPCR was performed in duplicate for each sample of three different experiments in keeping with the manufacturer’s instructions. All qPCR data were normalized to β-actin expression. Primers sequence, annealing temperature (Tm) and amplicon size for each gene are shown in Table S1.

### FACS analysis of glucose uptake

Control and fasentin-treated cells were washed twice with PBS supplemented with calcium and magnesium (DPBS), and then starved for 30 min with this DPBS. Cells were incubated for additional 30 min with DPBS supplemented with 5 mM glucose, 0.5 mM glutamine and 100 µM 2-NBDG (2-(N-(7-Nitrobenz-2-oxa-1,3-diazol-4-yl)Amino)-2-Deoxyglucose). Fasentin was present in all incubations. Relative glucose uptake was determined using a FACS VERSE^TM^ cytometer from BD Biosciences (San Jose, CA, USA) as previously described^55^. Data were analysed with BD FACSuite software.

### In vitro VEGFR2 kinase activity assay

VEGFR2 kinase activity was measured *in vitro* using the VEGFR2 (KDR) Kinase Assay kit (BPS Bioscience). Fasentin was tested at 100 µM according to the manufacturer’s instructions, determining the percentage of remaining kinase activity after 45 min incubation at 30 °C. 1 µM sunitinib was used as positive control of inhibition^24,25^. Data were collected using a GloMax-96 microplate luminometer (Promega).

### Western-blot

Cells treated or not with fasentin for 16 h were washed with cold PBS and lysed in RIPA buffer (50 mM Tris, pH 7.4, 150 mM NaCl, 1% Triton X-100, 0.25% sodium deoxycholate, 1 mM EDTA) containing phosphatase activity inhibitors (30 mM sodium fluoride, 1 mM sodium orthovanadate and 30 mM glycerophosphate) and protease activity inhibitors (cOmplete^TM^ Mini, Roche). Extracts were centrifuged at 13000 g for 5 min at 4 °C and evaluated for protein concentration using the Bradford method. Samples were heated for 5 min at 95 °C and separated on 10% polyacrylamide gels. Proteins were transferred to nitrocellulose membranes and blocked with 10% (w/v) semiskimmed dried milk. Blocked membranes were incubated overnight with primary antibodies (rabbit anti-phospho-p44/42 MAPK (ERK1/2) (Thr202/Tyr204) 1:1000, rabbit anti-p44/42 MAPK (ERK1/2) 1:2000, rabbit anti-phospho-Akt (Ser473) 1:250, rabbit anti-Akt 1:1500, or rabbit anti-vinculin 1:5000), washed, and later incubated with the peroxidase-linked secondary anti-rabbit antibody for 1 h at room temperature. Membranes were washed and finally incubated with the Supersignal^®^ West Pico chemiluminescent substrate system (Thermo Scientific). Image captions were taken with the ChemiDoc^TM^ XRS + System (Bio-Rad) using Image Lab^TM^ software. Densitometry analyses were made with Image J software and cropped blots were obtained using Adobe Photoshop CS6 software.

### Statistical analysis

Results are expressed as means ± SD. Statistical significance was determined using one-way or two-way ANOVA. When this analysis was not applicable, Student’s t-test analysis was performed. Values of p < 0.05 were considered to be statistically significant.

## Supplementary information

Supplementary information.
