# Supplementary material for: Fasentin diminishes endothelial cell proliferation, differentiation and invasion in a glucose metabolism-independent manner
Source: Sci Rep. 2020 Apr 9;10:6132. doi: 10.1038/s41598-020-63232-z (PMC7145862; doi:10.1038/s41598-020-63232-z)
Supplement: Supplementary file 1 — Supplementary information. [file 41598_2020_63232_MOESM1_ESM.pdf]

# Supplementary Information

## Fasentin diminishes endothelial cell proliferation, differentiation and invasion in a glucose metabolism-independent manner

**Mª Carmen Ocaña**[a,b]**, Beatriz Martínez-Poveda**[a,b]**, Manuel Marí-Beffa**[c]**, Ana R. Quesada**[a,b,d]**, Miguel Ángel Medina**[a,b,d*]

[a] Universidad de Málaga, Andalucía Tech, Departamento de Biología Molecular y Bioquímica, Facultad de Ciencias, E-29071 Málaga, Spain

[b] IBIMA (Biomedical Research Institute of Málaga), E-29071 Málaga, Spain

[c] Universidad de Málaga, Andalucía Tech, Departamento de Biología Celular, Genética y Fisiología, Facultad de Ciencias, E-29071 Málaga, Spain

[d] CIBER de Enfermedades Raras (CIBERER), E-29071 Málaga, Spain

*Corresponding author: Departamento de Biología Molecular y Bioquímica, Facultad de Ciencias, Universidad de Málaga, E-29071 Málaga, Spain. Fax: +34 952131674.

E-mail address: medina@uma.es (M.Á. Medina).

Supplementary Table S1. Primers used for qPCR.

| Gene | Primers | Annealing temperature (ºC) | Amplicon size (bp) |
|---|---|---|---|
| β-actin | Fw: GACGACATGGAGAAAATCTG<br>Rv: ATGATCTGGGTCATCTTCTC | 60 | 131 |
| MMP-2 | Fw: GACATACATCTTTGCTGGAGAC<br>Rv: ACGCTCTTCAGACTTTGGTTCT | 60 | 207 |
| TIMP-1 | Fw: CACCTTATACCAGCGTTATG<br>Rv: TTTCCAGCAATGAGAAACTC | 60 | 168 |
| TIMP-2 | Fw: GGCCTGAGAAGGATATAGAG<br>Rv: CTTTCCTGCAATGAGATATTCC | 60 | 104 |
| TIMP-3 | Fw: CTGACAGGTCGCGTCTATGATG<br>Rv: AGCAAGGCAGGTAGTAGCAG | 60 | 157 |
| TIMP-4 | Fw: AAATCTCCAGTGAGAAGGTAG<br>Rv: TCTCAAACCCTTTGAACATC | 60 | 105 |
| uPA | Fw: CGCCACACACTGCTTCATG<br>Rv: CCCCTTGCGTGTTGGAGTT | 60 | 89 |
| uPAR | Fw: GCCCAATCCTGGAGCTTGA<br>Rv: TCCCCTTGCAGCTGTAACACT | 60 | 63 |
| PAI-1 | Fw: GCACAACCCCACAGGAACA<br>Rv: GTCCCGATGAAGGCGTCTTT | 60 | 80 |

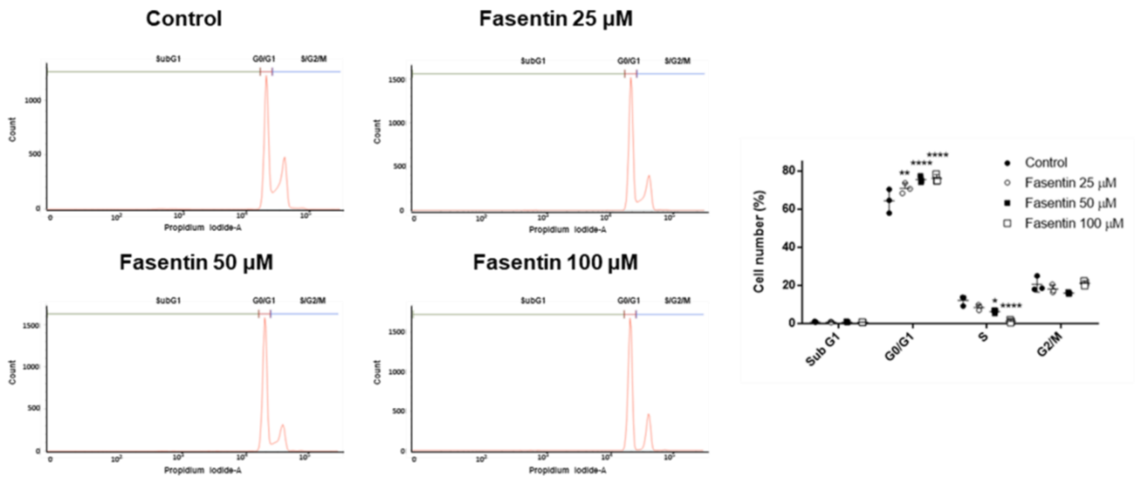


Supplementary Figure S1. Effect of fasentin on endothelial cell cycle distribution. HMECs were exposed for 16 h to fasentin at the indicated concentrations, stained with propidium iodide and percentages of cells on subG1, G1, S and G2/M phases were determined using a FACS VERSETM cytometer. A representative result and the calculated values for cell subpopulations, expressed as means ± SD of three independent experiments, are shown. *$p < 0.05$, **$p < 0.01$, ****$p<0.0001$ versus untreated control.

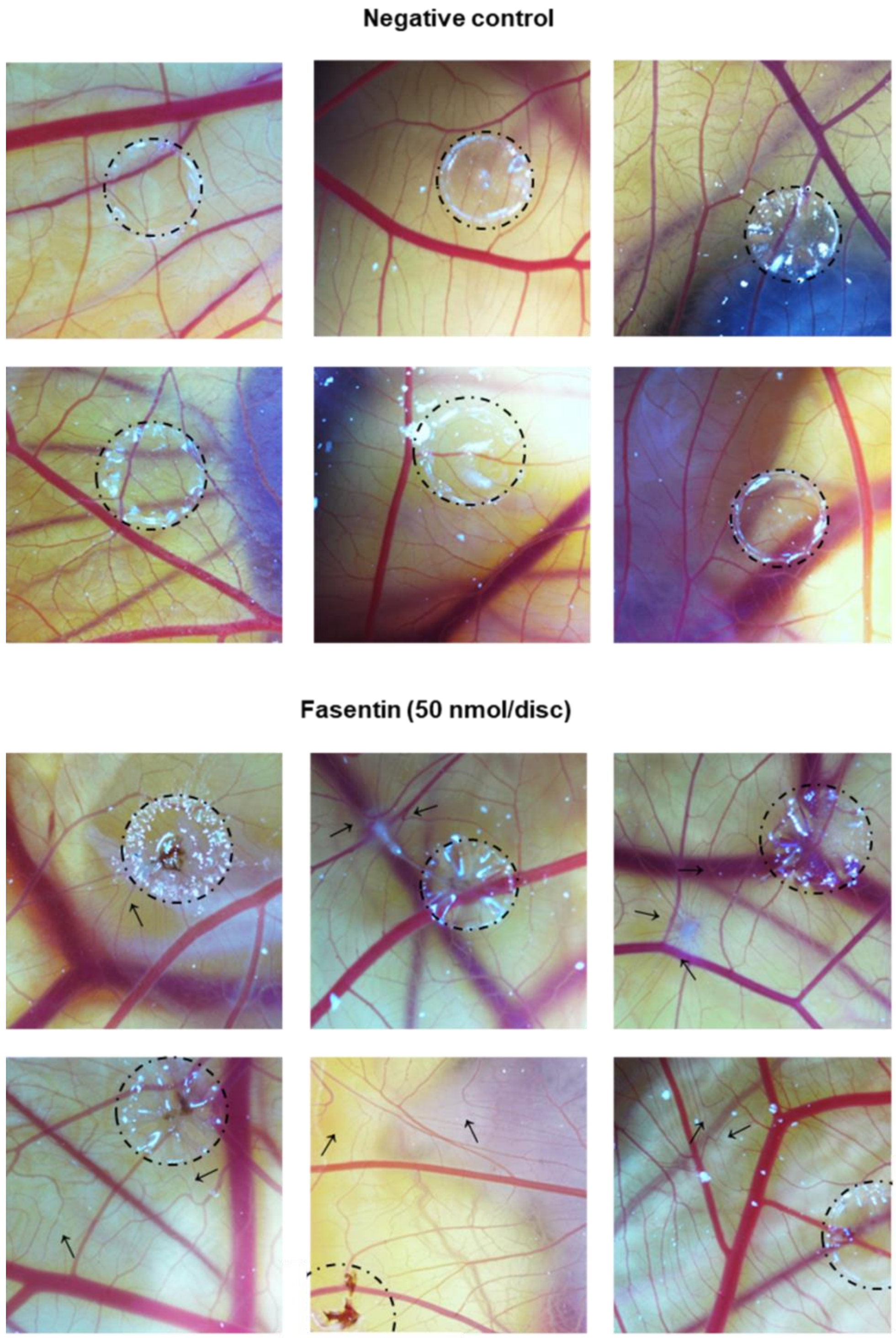


Supplementary Figure S2. Additional photographs from the results of the CAM assay.

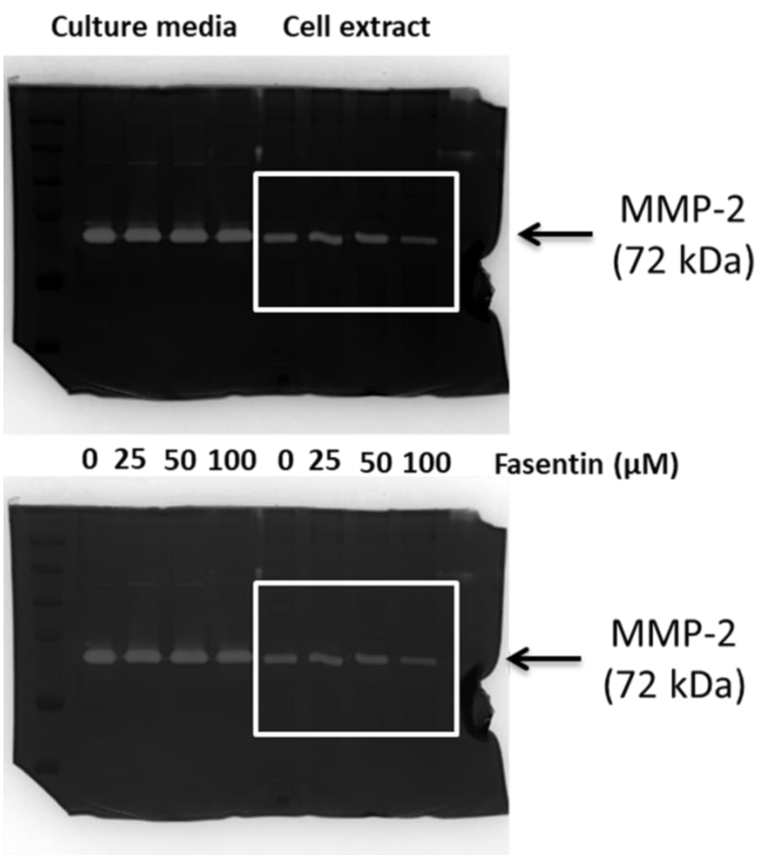


Supplementary Figure S3. Uncropped blots corresponding to Figure 6a with two different expositions.

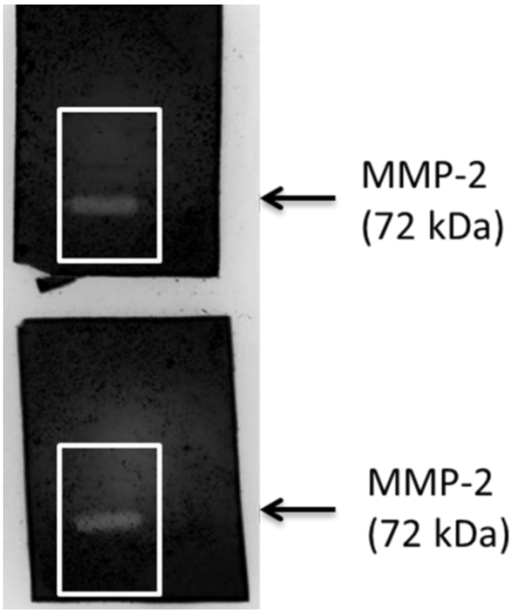


Supplementary Figure S4. Uncropped blots corresponding to Figure 6c.

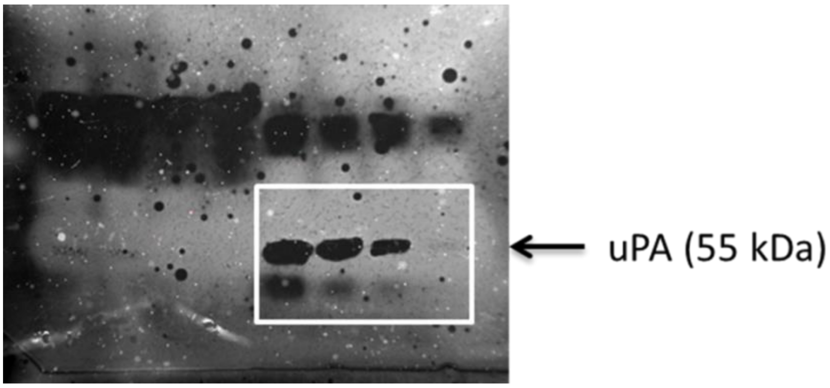


Supplementary Figure S5. Uncropped blot corresponding to Figure 6d.

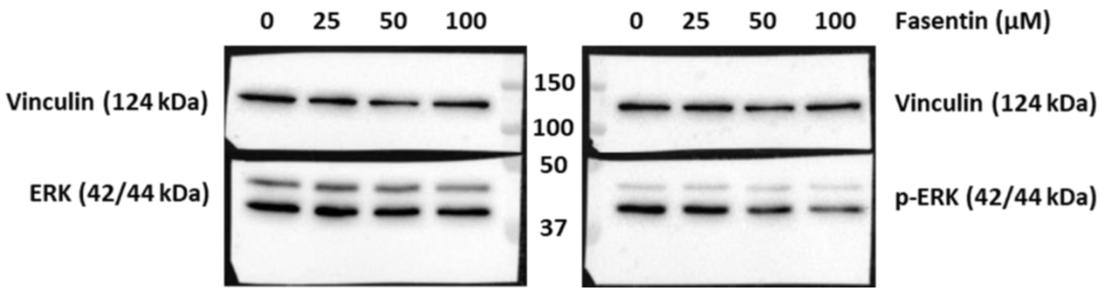


Supplementary Figure S6. Uncropped blots corresponding to Figure 8a.

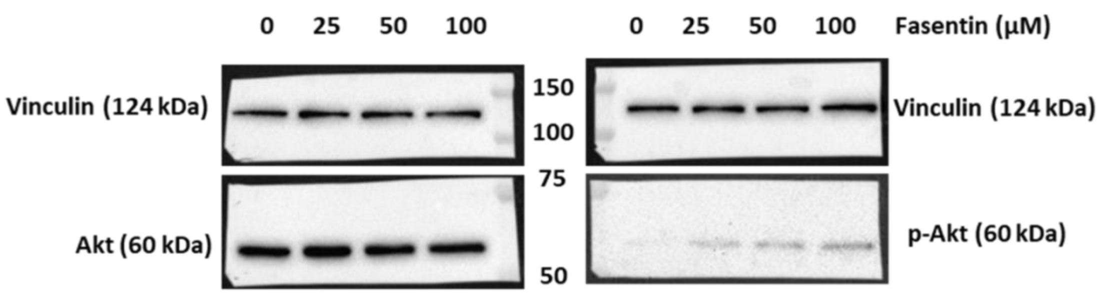


Supplementary Figure S7. Uncropped blots corresponding to Figure 8b.
